# ​​Breaking the Bowel: A Subacute Ischemic Complication of Chronic Methamphetamine Use

**DOI:** 10.7759/cureus.98331

**Published:** 2025-12-02

**Authors:** Benjamin Moran, Tiffany Scotto, Sophia Sheikh

**Affiliations:** 1 Emergency Medicine, University of Florida College of Medicine – Jacksonville, Jacksonville, USA; 2 Internal Medicine, University of Florida College of Medicine – Jacksonville, Jacksonville, USA

**Keywords:** abdominal abscess, bowel ischemia, emergency exploratory laparotomy, fluid resuscitation, mesenteric ischemia, methamphetamine, necrotic bowel, sepsis, substance use disorder, toxicology

## Abstract

Methamphetamine is a potent sympathomimetic agent primarily associated with cardiovascular and neuropsychiatric effects. However, its gastrointestinal complications, particularly ischemic bowel disease, are under-recognized in emergency medicine literature.

A 34-year-old man with a history of chronic methamphetamine use presented to the emergency department (ED) seeking medical clearance for rehabilitation admission. He also reported mild, diffuse abdominal pain persisting for three days. Vital signs revealed tachycardia and hypotension. Physical examination showed mild subjective diffuse abdominal tenderness without peritoneal signs. Laboratory studies indicated leukocytosis, elevated lactate, and acute kidney injury. Non-contrast abdominal and pelvic computed tomography (CT) demonstrated pneumoperitoneum and extensive mesenteric stranding consistent with ischemia. The patient was managed with intravenous fluids, broad-spectrum antibiotics, and emergent surgical consultation. Exploratory laparotomy revealed hemoperitoneum, a large abscess cavity, necrosis of the cecum, and mesenteric ischemia. A resection of the cecum and terminal ileum was performed. Postoperatively, the patient required intensive care management for septic shock and renal failure but eventually recovered and was discharged after 15 days.

This case underscores the importance of considering chronic methamphetamine use as a potential etiology for gastrointestinal ischemia and sepsis, even in patients presenting with subtle abdominal complaints. Early recognition and intervention are crucial for improving outcomes in this high-risk population.

## Introduction

The most common side effects and complications of methamphetamine use include acute symptoms such as chest pain, palpitations, hypertension, hyperthermia, impulsivity, psychosis, and delirium [1]. However, its impact on the gastrointestinal system, particularly in causing ischemic bowel disease, remains underappreciated in emergency medicine literature compared to other stimulants such as cocaine [2-4]. The vasoconstrictive properties of methamphetamine can lead to mesenteric ischemia, a condition that, if not promptly diagnosed and treated, carries a high mortality rate [5].

Mesenteric ischemia, bowel obstruction, and sepsis can evolve insidiously in the context of chronic methamphetamine use, often leading to delayed diagnosis and poor outcomes. Emergency physicians are uniquely positioned to identify these presentations early, especially in patients who may not initially appear acutely ill. This report highlights an unusual case of subacute non-occlusive ischemic bowel disease from chronic methamphetamine use. Additionally, this case also aims to raise awareness of chronic methamphetamine use as a risk factor for serious gastrointestinal pathology and to reinforce the need for high suspicion and early surgical consultation in such patients. This is especially important given the fact that chronic methamphetamine use rose 45% among United States adults from 2015 to 2019 [6].

## Case presentation

A 34-year-old man with a history of chronic methamphetamine use presented to the ED in October 2024, requesting medical clearance for admission to a rehabilitation facility for assistance with his methamphetamine use disorder. He also reported mild, diffuse abdominal pain for the past three days. He admitted to daily methamphetamine use over the past several weeks but denied nausea, vomiting, fever, decreased appetite, decreased fluid intake, or changes in bowel habits. Of note, the patient denied co-ingestion of alcohol or other xenobiotics. He had no prior surgical history.

Upon arrival, the patient was tachycardic at 112 beats per minute, diaphoretic, and appeared mildly uncomfortable and paranoid, which he attributed to his methamphetamine use. He was hypotensive at 89/64 mm Hg with preserved mean arterial pressures (72 mm Hg) and remained afebrile. Abdominal examination revealed diffuse subjective mild tenderness without rebound or guarding. There were no signs of peritonitis, though his general discomfort appeared disproportionate to the abdominal examination findings. He had decreased bowel sounds. Pulses in all four extremities were equal and he exhibited no focal neurological findings.

Laboratory studies were notable for a white blood cell count of 14.88 x10^3/μL, a rising lactate level (initially 2.2 mmol/L, peaking at 3.7 mmol/L), and acute kidney injury (creatinine of 7.88 mg/dL elevated from 0.97 mg/dL one year prior), and he remained anuric in the ED without any output into his Foley catheter (Table 1).

**Table 1:** Pertinent laboratory findings. The table shows selected pertinent laboratory studies, including complete blood count, basic metabolic panel, lactic acid, and toxicology screen from the date of presentation compared to one year prior. Abnormal values demonstrate acute kidney injury, metabolic acidosis, leukocytosis, and methamphetamine exposure. Abnormal values are reported relative to the reference range. WNL: Within Normal Limits; H: High; L: Low, Not Obtained: Not Collected at the Time of Presentation *: repeat value collected three hours after the initial presentation

Test	Reference Range	One Year Prior	Date of Presentation
WBC (×10³/µL)	4.0 – 11.0	5.81 (WNL)	14.78 (H)
Bands (%)	0 – 10	0.9 (WNL)	28.6 (H)
Sodium (mmol/L)	135 – 145	137 (WNL)	132 (L)
Potassium (mmol/L)	3.5 – 5.0	4.9 (WNL)	5.2 (H)
Chloride (mmol/L)	98 – 106	99 (WNL)	87 (L)
CO₂ (mmol/L)	22 – 29	30 (H)	18 (L)
BUN (mg/dL)	7 – 20	9 (WNL)	77 (H)
Creatinine (mg/dL)	0.6 – 1.3	0.97 (WNL)	7.88 (H)
eGFR (mL/min/1.73m²)	>60	76 (WNL)	9 (L)
Lactic acid (mmol/L)	0.5 – 2.0	Not Obtained	2.2 (H), 3.7* (H)
Urine drug screen (immunoassay)	Negative	Not Obtained	Methamphetamine (Positive)

**Figure 1:**
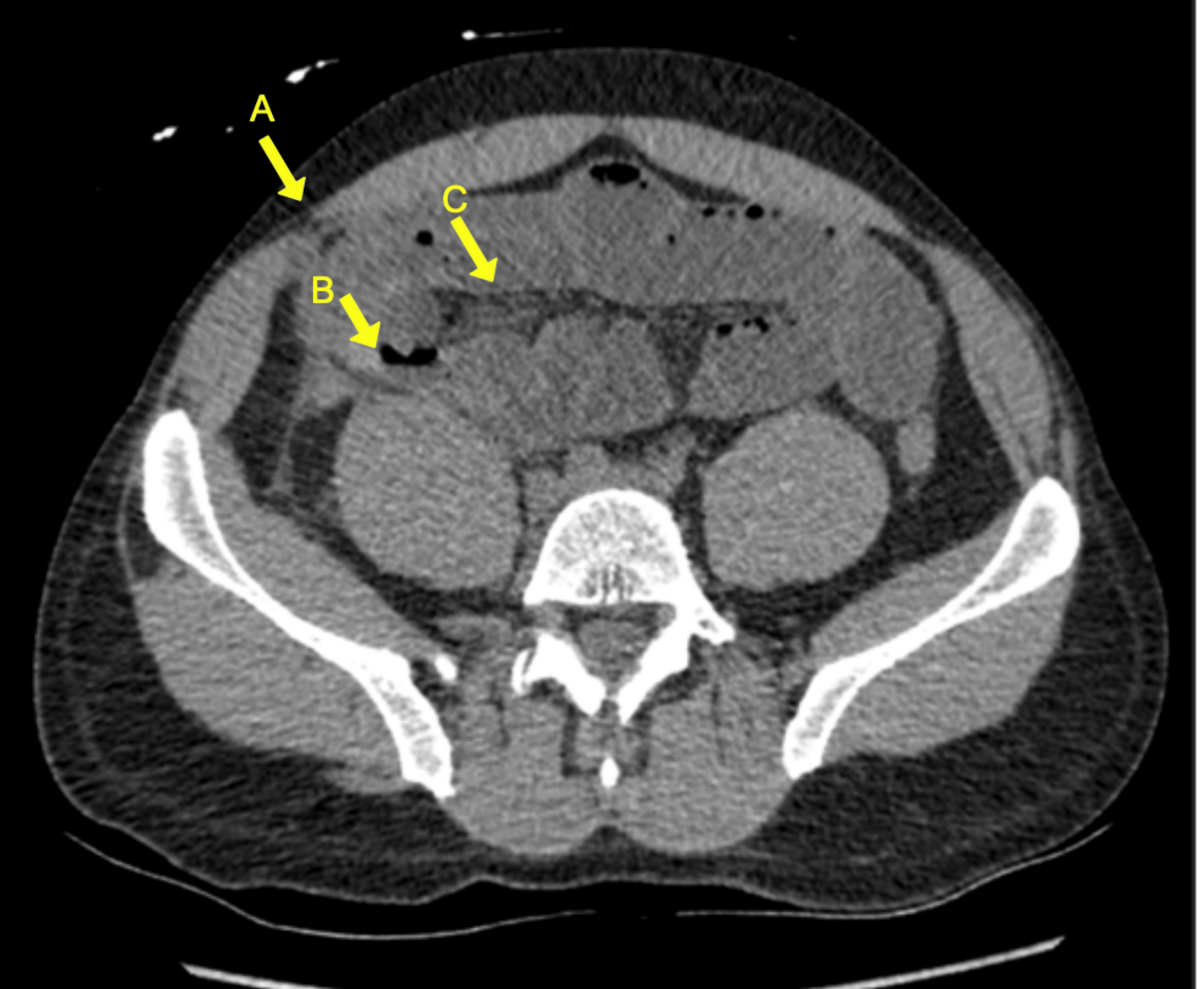
Abdominal computed tomography (CT) scan. Axial non-contrast CT of the abdomen demonstrates pneumoperitoneum (A), dilated fluid-filled small bowel loops (B), and mesenteric stranding (C). These findings are concerning for bowel ischemia with perforation, later confirmed intraoperatively.

A non-contrast CT scan of the abdomen and pelvis revealed pneumoperitoneum, a pelvic abscess measuring 6.8 x 3.3 cm, dilated and fluid-filled small bowel loops concerning for developing small bowel obstruction, and extensive mesenteric stranding consistent with ischemia (Figure 1).

Given the concern for sepsis and bowel ischemia, the patient received 2.5 liters of intravenous fluid and was started on vancomycin and piperacillin-tazobactam. Due to the progression of his clinical picture, including hypotension, rising lactate, and persistent abdominal pain, he was transferred to the surgical intensive care unit for emergent surgical intervention. 

Exploratory laparotomy revealed hemoperitoneum with foul-smelling fluid and a large abscess cavity in the right lower quadrant. There was necrosis of the cecum and signs of mesenteric ischemia. The surgical team performed an ileocecectomy, removing the necrotic right colon and distal ileum. Due to coagulopathy and clinical instability in the setting of septic shock and acidosis, the abdomen was left in discontinuity with a vacuum-assisted closure system in place.

Postoperatively, the patient remained critically ill with ongoing septic shock and renal failure. He required vasopressor support and continued on the same broad-spectrum antibiotics. His abdominal wound was managed with serial dressing changes and several re-explorations. Despite the severity of his illness, he showed gradual clinical improvement with intensive supportive care and his renal function returned to baseline without renal replacement therapy. He was eventually discharged to a rehabilitation facility for assistance with his methamphetamine use disorder after 15 days in the surgical intensive care unit.

## Discussion

Methamphetamine exerts potent sympathomimetic effects that induce systemic vasoconstriction, predisposing multiple organ systems, including the gastrointestinal tract, to ischemia [7,8]. Experimental animal studies further support this mechanism, demonstrating methamphetamine-related intestinal inflammation, impaired mucosal integrity, and microvascular injury over time [9-14]. Although the cardiovascular, psychiatric, and neurologic complications of methamphetamine use are well characterized, methamphetamine-associated bowel ischemia remains relatively uncommon in the literature. Most published cases involve an abrupt onset of severe abdominal pain following acute intoxication or binge use, often in individuals without consistent, long-term daily exposure [15,16].

In contrast to prior reports, our patient was a chronic, daily user who developed symptoms gradually over several days. This more insidious presentation aligns with the possibility of partial or intermittent mesenteric hypoperfusion, allowing ischemia to evolve slowly into necrosis and abscess formation rather than producing the rapid, catastrophic ischemia described in binge-use cases. The absence of peritoneal signs in this patient likely contributed to diagnostic delay, emphasizing the challenge of identifying mesenteric ischemia when exam findings are subtle. Therefore, this case represents a novel presentation in emergency medicine literature.

The distinction between chronic and binge use may also explain the divergent clinical course. Chronic vasoconstriction may lead to progressive microvascular compromise with temporary periods of preserved perfusion due to small bowel collateral circulation [17]. This contrasts with binge-induced ischemia, where sudden, profound vasospasm overwhelms collateral flow and results in abrupt, severe ischemic insult [18]. Our patient had no other risk factors, no prior abdominal surgeries, immunocompromise, or other xenobiotic exposures, making methamphetamine the most plausible driver of his vascular pathology.

This case highlights the need for emergency physicians to maintain vigilance when evaluating methamphetamine users with vague abdominal complaints. Even mild or atypical symptoms may mask evolving ischemia, and early imaging should be strongly considered when laboratory abnormalities such as leukocytosis or lactic acidosis are present.

## Conclusions

Early recognition of mesenteric ischemia is critical in chronic methamphetamine users, as symptoms may be subtle and nonspecific. This case demonstrates that abdominal pathology can be easily overlooked, especially when abdominal pain is not the main chief complaint, underscoring the importance of a thorough history and physical examination. Prompt imaging, aggressive resuscitation, broad-spectrum antibiotics, and early surgical consultation were essential to this patient’s survival. Emergency physicians should maintain a high index of suspicion for ischemic bowel in methamphetamine users presenting with shock, lactic acidosis, or abdominal pain out of proportion to physical examination findings.

## References

[1] Leyde S, Tilhou AS, Tsui JI (2025). Methamphetamine use disorder. JAMA.

[2] Holubar SD, Hassinger JP, Dozois EJ, Masuoka HC (2009). Methamphetamine colitis: a rare case of ischemic colitis in a young patient. Arch Surg.

[3] Johnson TD, Berenson MM (1991). Methamphetamine-induced ischemic colitis. J Clin Gastroenterol.

[4] Elramah M, Einstein M, Mori N, Vakil N (2012). High mortality of cocaine-related ischemic colitis: a hybrid cohort/case-control study. Gastrointest Endosc.

[5] Blaser AR, Mändul M, Björck M (2024). Incidence, diagnosis, management and outcome of acute mesenteric ischaemia: a prospective, multicentre observational study (AMESI Study). Crit Care.

[6] Han B, Compton WM, Jones CM, Einstein EB, Volkow ND (2021). Methamphetamine use, methamphetamine use disorder, and associated overdose deaths among US adults. JAMA Psychiatry.

[7] Citron BP, Halpern M, McCarron M (1970). Necrotizing angiitis associated with drug abuse. N Engl J Med.

[8] Smith SW, Goldfrank LR, Hoffman RS (2019). Goldfrank's Toxicologic Emergencies. 11th ed.

[9] Chen LJ, Zhi X, Zhang KK (2021). Escalating dose-multiple binge methamphetamine treatment elicits neurotoxicity, altering gut microbiota and fecal metabolites in mice. Food Chem Toxicol.

[10] Davidson M, Mayer M, Habib A (2022). Methamphetamine induces systemic inflammation and anxiety: the role of the gut-immune-brain axis. Int J Mol Sci.

[11] Shen S, Zhao J, Dai Y, Chen F, Zhang Z, Yu J, Wang K (2020). Methamphetamine-induced alterations in intestinal mucosal barrier function occur via the microRNA-181c/ TNF-α/tight junction axis. Toxicol Lett.

[12] Ginsburg M, Obara P, Lambert DL (2018). ACR Appropriateness Criteria(®) imaging of mesenteric ischemia. J Am Coll Radiol.

[13] Wang LB, Xu LL, Chen LJ (2022). Methamphetamine induces intestinal injury by altering gut microbiota and promoting inflammation in mice. Toxicol Appl Pharmacol.

[14] Zhao J, Shen S, Dai Y, Chen F, Wang K (2019). Methamphetamine induces intestinal inflammatory injury via Nod-like receptor 3 protein (NLRP3) inflammasome overexpression in vitro and in vivo. Med Sci Monit.

[15] Garzelli L, Ben Abdallah I, Nuzzo A (2023). Insights into acute mesenteric ischaemia: an up-to-date, evidence-based review from a mesenteric stroke centre unit. Br J Radiol.

[16] Fitzpatrick LA, Rivers-Bowerman MD, Thipphavong S, Clarke SE, Rowe JA, Costa AF (2020). Pearls, pitfalls, and conditions that mimic mesenteric ischemia at CT. Radiographics.

[17] Clair DG, Beach JM (2016). Mesenteric ischemia. N Engl J Med.

[18] Anderson JE, Brown IE, Olson KA, Iverson K, Cocanour CS, Galante JM (2018). Nonocclusive mesenteric ischemia in patients with methamphetamine use. J Trauma Acute Care Surg.

